# A synergistic herbal formulation targeting *Malassezia furfur* and *Staphylococcus epidermidis* for effective dandruff management

**DOI:** 10.3389/fmicb.2025.1654658

**Published:** 2025-11-21

**Authors:** Satyendra Pratap Singh, Bhanu Kumar, Ankita Misra, Poonam Rawat, Deepali Tripathi, Sharad Srivastava

**Affiliations:** 1 Division of Pharmacognosy, CSIR- National Botanical Research Institute, Lucknow, India; 2 Department of Postharvest Science of Fresh Produce, Institute for Postharvest and Food Sciences, Agricultural Research Organization (ARO), Volcani Center, Rishon LeZion, Israel; 3 FEST Division, CSIR- Indian Institute of Toxicology Research, Lucknow, India

**Keywords:** *Malassezia furfur*, *Staphylococcus epidermidis*, dandruff, *Eucalyptus citriodora*, phytomolecules

## Abstract

Dandruff is a chronic scalp condition and the mildest form of seborrheic dermatitis, associated with microbial dysbiosis primarily involving *Malassezia furfur* and *Staphylococcus epidermidis*. Conventional anti-dandruff treatments are often limited by recurrence and reliance on synthetic antifungal agents. This study aimed to develop and evaluate a synergistic, oil-based herbal formulation with antimicrobial activity against dandruff-causing microorganisms. *The herbal constituent, Eucalyptus citriodora (EC) oil, individually signifies its efficacy against key dandruff-causing microbes,* while *Centella asiatica* and *Wedelia trilobata* were included for their complementary bioactive and skin-healing properties. A total of three formulations (F1, F2, and F3), containing 8, 10, and 12% EC oil, respectively, were tested for antimicrobial efficacy. The optimized formulations (F2 and F3) exhibited significant antimicrobial activity, resulting in 1.8–1.9-fold and 2.0–2.5-fold reductions in *M. furfur* and *S. epidermidis* cell viability, respectively. Fluorescence-based LIVE/DEAD viability assays and scanning electron microscopy (SEM) confirmed membrane disruption and structural damage in the treated cells of *M. furfur*. In addition, molecular docking and dynamics simulations demonstrated strong binding affinities of *Eucalyptus citriodora* oil constituents toward lanosterol 14α-demethylase (LDM), suggesting disruption of the ergosterol biosynthesis pathway as a plausible antifungal mechanism. The obtained findings indicated that the formulated herbal combination exerts a multi-targeted antimicrobial effect and represents a promising natural alternative for controlling microbial populations involved in dandruff pathogenesis.

## Graphical abstract

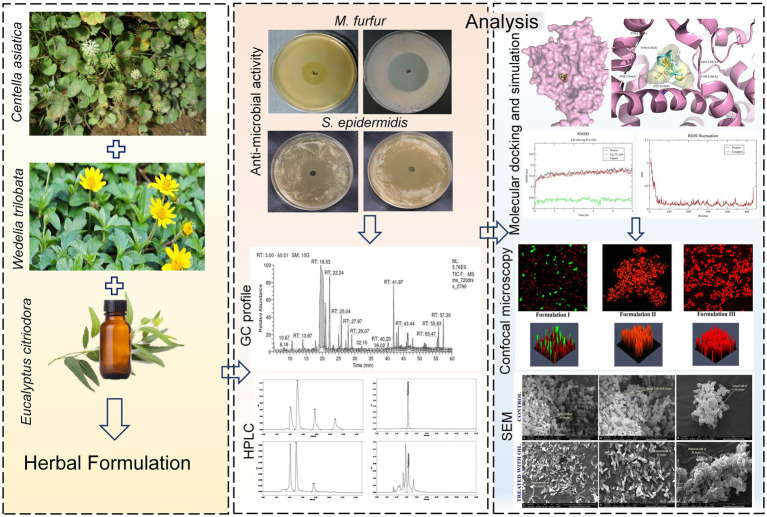

## 1 Introduction

Dandruff, a prevalent scalp disorder, manifests as flaky white to yellowish scales and can progress to infections in areas such as the eyebrows and behind the ears, accompanied by itchy, flaking skin without visible inflammation (Chaiyana et al., 2022). It represents the mildest form of seborrheic dermatitis, affecting nearly half of the global population (Grimshaw et al., 2019; Newton-Fenner et al., 2025; Yu et al., 2025) and impacting over 50% of adults at some stage (Sowell et al., 2022; Polaskey et al., 2024). This condition imposes a significant economic burden, with annual expenditures exceeding $300 million on over-the-counter dandruff products (Güner et al., 2021; Alsmeirat et al., 2022; Jain et al., 2022). Dandruff’s etiology involves both non-microbial and microbial factors, along with genetic predispositions. Non-microbial factors include scalp damage, susceptibility to oleic acid, and dry scalp conditions, which can eventually lead to microbial factors (Borda and Wikramanayake, 2015). The causal organism of dandruff, *Malassezia* sp., is the primary microbial factor, whose pathogenicity can be influenced by stress, fatigue, weather extremes, immunosuppression, and neurological disorders of the host (Rudramurthy et al., 2014). In addition, bacteria such as *Staphylococcus epidermidis* and *Propionibacterium acnes* also contribute to dandruff onset (Clavaud et al., 2013; Xu et al., 2016).

Current therapeutic strategies for dandruff rely heavily on synthetic antifungal agents (e.g., ketoconazole), keratolytics, and antimicrobial agents such as zinc pyrithione, selenium sulfide, and imidazole derivatives (Sharma et al., 2013). Moreover, chemical-based oil formulations are relatively less, and many synthetic products are marketed as shampoo. However, prolonged use of such agents has been associated with adverse effects including irritation, dryness, and the potential for resistance (Güner and İlhan, 2020). Despite various chemical-based anti-dandruff market products, effectively suppressing *Malassezia* sp. and preventing dandruff recurrence remains challenging. Consequently, there is a growing interest in developing plant-derived therapeutics, which offer safer, sustainable, and effective alternatives.

Medicinal plants have been recognized for their therapeutic properties in traditional systems of medicine. In Ayurveda, the use of numerous botanicals for treating a wide range of diseases and disorders has been long documented (Patwardhan, 2000). These claims have been validated through modern analytical tools and *in vivo* and clinical studies (Mukherjee et al., 2017). Medicinal plants such as *Aloe vera*, *Eclipta alba*, *Wedelia trilobata*, *Eucalyptus* sp., *Sapindus* sp., and *L. inermis* have been used for their benefits in hair care (Gupta et al., 2010).

*Centella asiatica*, from the Apiaceae family, is traditionally used in Southeast Asia for treating skin diseases and inflammation (Yu et al., 2007). Asiaticoside, its main active constituent, shows significant wound-healing activity (Shukla et al., 1999). In a study on hair growth, the titrated extract of *C. asiatica* increased the sphere size of 3D spheroid-cultured human dermal papilla cells and enhanced hair-growth-related genes (Choi et al., 2017). Similarly, the genus *Wedelia* (Asteraceae), known as ‘Peet Bhringraj,’ is used for skin diseases and alopecia. Its leaves are used for dyeing grey hair, promoting hair growth, and treating alopecia (Nithin Manohar et al., 2011). Essential oil of *Eucalyptus citriodora* (EC) has been shown to possess significant antifungal, anticandidal, and antibacterial activities (Ramezani et al., 2002; Li et al., 2022; El-Sakhawy et al., 2023). Its strong antioxidant activity is attributed to monoterpenoids such as citronellal (Singh et al., 2012).

This study aims to formulate and scientifically validate a synergistic plant-based herbal blend and assess its efficacy against dandruff-causing microorganisms. Mechanistic insights were explored using *in silico* molecular docking and dynamics simulations targeting fungal ergosterol biosynthesis enzymes, while structural alterations in treated microbial cells were examined through confocal and scanning electron microscopy. The findings suggest that the developed formulation is an effective plant-based alternative for managing dandruff and preventing its recurrence by reducing dandruff-causing microorganisms.

## 2 Materials and methods

### 2.1 Experimental materials

The experimental setups, raw materials of the selected herbal leads, extraction protocols, and dandruff-causing microbes incorporated in the present investigation are detailed in the Supplementary material.

### 2.2 Pre-formulation studies

#### 2.2.1 Antimicrobial activity of the selected herbal leads

The antimicrobial activity of *Centella asiatica*, *Wedelia trilobata,* and *Eucalyptus citriodora* oil (EC oil) was evaluated using the agar well diffusion assay (Magaldi et al., 2004) with minor modifications. Briefly, crude extracts of *C. asiatica* and *W. trilobata* were dissolved in DMSO to obtain final concentrations of 20, 40, 60, and 80 mg mL^−1^, while EC oil was prepared at concentrations ranging from 3 to 14% (v/v). A 100 μL aliquot of freshly prepared broth cultures of *Malassezia furfur* and *Staphylococcus epidermidis* was spread uniformly on the surface of Dixon’s agar (Himedia, India) and nutrient agar (Himedia, India) plates, respectively. Thereafter, 6 mm-diameter wells (as per the experimental design) were aseptically created with the help of a sterile cork borer. Different concentrations (abovementioned) of the selected herbal extracts and oil (100 μL) were then aseptically added to the wells, and the plates were kept in a laminar flow hood until the plant extracts were completely diffused into the wells. Plates inoculated with *M. furfur* were incubated at 30 ± 2 °C for 5–7 days to allow visible lawn formation, whereas those inoculated with *S. epidermidis* were incubated at 28 ± 2 °C for 24 h. The diameter (in mm) of inhibition zones around the well was measured to evaluate the antimicrobial efficacy of herbal leads. All assays were conducted in triplicate, and mean inhibition values were recorded.

#### 2.2.2 Preparation of different formulations from the herbal ingredients

The extracts of *C. asiatica*, *W. trilobata,* and EC oil were blended in specified ratios (based on primary screening) to prepare formulation 1 (3% *C. asiatica,* 4% *W. trilobata*, 8% EC oil), formulation 2 (5% *C. asiatica,* 3% *W. trilobata*,10% EC oil), and formulation 3 (5% *C. asiatica,* 4% *W. trilobata*,12% EC oil).

### 2.3 Post-formulation studies

#### 2.3.1 Anti-dandruff activity of the formulated oil

The antifungal efficacy of the formulated oil, comprising a blend of the three herbal components, was similarly assessed using the agar well diffusion assay. In addition, different strategic experiments were executed to further examine the efficacy of the different combinations of the formulated oil.

#### 2.3.2 Preparation of microbial inoculum and treatments

Dandruff-causing microbes, such as *M. furfur* and *S. epidermidis,* were separately cultured in Sabouraud Dextrose Broth (SDB) and Nutrient Broth (NB), followed by incubation at 30 ± 2 °C (for *M. furfur*) and 28 ± 2 °C (for *S. epidermidis*) under their respective growth conditions. Spores/cells of microbial growth were harvested through centrifugation at 12000 rpm for 10 min, washed twice in saline (0.8%NaCl), and re-suspended to achieve the standardized inoculum densities of 2×10^5^ spores/cells ml^−1^ (*M. furfur*) and 1×10^8^ CFU ml^−1^ (*S. epidermidis*), respectively.

The treatments were as follows: formulations 1, 2, and 3 (F1, F2, and F3; different permutation combinations of the selected herbal leads), along with two anti-fungal standards (Standard 1: 50 mg ketoconazole; Standard 2; 50 mg fluconazole) and two commercially available herbal anti-dandruff market products (MP1 and MP2) containing plant-derived components such as rosemary, tea tree oil, neem, and other herbal extracts, which are commonly claimed to exhibit anti-dandruff activity, and a control without treatment.

##### 2.3.2.1 Assessment of average cell count-based anti-dandruff activity

To further assess the antifungal efficacy of the formulations, average cell counts were determined following treatment. A non-treated spore suspension (500 μL) of standardized dandruff-causing microorganisms (*M. furfur* and *S. epidermidis*) was aseptically dispensed into 1 mL of freshly prepared SDB and NB (1 mL), supplemented with 100 μL of the different concentrations of the tested formulations (Formulation 1, 2 and 3), along with the respective control, standards, and market products (as per treatment) in culture tubes. Subsequently, the experimental setup was incubated at 30 ± 2 °C and 28 ± 2 °C for *M. furfur* and *S. epidermidis,* respectively. Furthermore, a 100 μL aliquot was sampled (after every 24 h) from each treatment for quantitative assessment. *S. epidermidis* growth under the different treatments was estimated by plating serial decimal dilutions (100 μL) on NA, followed by incubation at 28 ± 2 °C. The *S. epidermidis* colonies on NA plates were recorded to determine the average bacterial cell count (Korte et al., 2023). In addition, the average yeast cell count of *M. furfur* was determined using a hemocytometer (Sonderegger et al., 2018). All experiments were performed in triplicate, and the results were expressed as CFU mL^−1^ for *S. epidermidis* and cells mL^−1^ for *M. furfur*.

##### 2.3.2.2 Microplate assay-based anti-dandruff activity

The antifungal activity of the formulations was additionally evaluated using a microplate-based cell viability assay. Freshly grown dandruff-causing inocula (500 μL) of *M. furfur* and *S. epidermidis* were dispensed aseptically into different culture tubes. Subsequently, 100 μL of the different treatments was homogenized with the dispensed inocula of the dandruff-causing microbes separately (as per the treatments). The experimental setup was incubated at 30 ± 2 °C (for *M. furfur*) and 28 ± 2 °C (for *S. epidermidis*) under optimal growth conditions.

The *in vitro* antimicrobial activity of the developed formulations was further assessed using a bacterial/yeast cell viability test through cytofluorometric assay. Post-incubation, a 100 μL aliquot of the treated cells of the dandruff-causing organisms was stained with fluorescein diacetate (FDA; 2 mg/mL) for 10 min at room temperature to detect metabolically active cells. The stained cells were washed three times with phosphate-buffered saline (PBS; pH 7.0) for complete elimination of excess stain and culture media supplements. Subsequently, the fluorescence intensity of FDA was measured using a microplate reader (Tecan, Infinite 200 PRO, Switzerland) at excitation and emission wavelengths of 488 and 515 nm, respectively. Viability was calculated based on relative fluorescence intensity, and all measurements were performed in triplicate.

#### 2.3.3 QC parameters of the formulated oil

Different parameters of the selected herbal ingredients in the formulated oil were evaluated as per guidelines (International, 2005; WHO, 2007; Medicine and Homoeopathy, 2008). The obtained data are tabulated in Supplementary Tables S1, S2.

#### 2.3.4 Metabolite profiling/QC parameters of the formulated oil

##### 2.3.4.1 Assessment of major metabolites in *C. asiatica* and *W. trilobata* through RP-HPLC-PDA

###### 2.3.4.1.1 Standard and sample preparation

The stock solutions of centelloside standards, i.e., asiatic acid (AA), madecassic acid (MA), asiaticoside (AS), madecassoside (MS), and wedelolactone (WDL), were prepared by dissolving 10 mg in 1 mL of high-performance liquid chromatography (HPLC)-grade methanol and stored at 4 °C until analysis. A working solution of 1 mg/mL was prepared by a tenfold dilution of the stock solution with HPLC-grade methanol. Plant samples were prepared by reconstituting 10 mg of the extract in methanol to obtain a final concentration of 10 mg/mL. The working dilutions of the standards and samples were duly filtered through a syringe filter (0.45 μm, Millipore, Pall, USA) prior to analysis.

###### 2.3.4.1.2 Chromatographic conditions

The quantification of the *C. asiatica* and *W. trilobata* extracts was performed using a reverse-phase HPLC (RP-HPLC) system (Waters, Massachusetts, USA). The separation of the plant extract and standards was achieved on a C_18_ RP-HPLC column (4.6 × 250 mm internal diameter, Massachusetts, USA). For centellosides AA, MA, AS, and MS, the mobile phase used was water (A) and acetonitrile (B) (40:60, v/v, A: B) with a flow rate of 0.5 mL min^−1^, thermostatted at 35 °C for a total run time of 15 min. The injection volume was 2 μL for the plant extract and 5 μL for the standards. For WDL, the isocratic elution program was standardized following the protocol established by Patil et al. (2014), with slight modifications. The analysis was performed using a binary solvent system consisting of methanol and 0.5% acetic acid in water (55:45 v/v), and the total run time was 20 min. The centellosides and WDL were scanned at 195 nm and 254 nm, respectively. Identification of peaks was performed by comparing the R_t_ of the standards with that of the sample peaks and by comparing absorption spectra of the standards with those of the samples over the range of 190-400 nm. The amount of individual metabolites in the plant extract was calculated with respect to the linear regression curve of the standard concentrations versus peak area and was expressed in μg mg^−1^.

##### 2.3.4.2 Metabolite profiling of *Eucalyptus citriodora* essential oil

The qualitative and quantitative analysis of the oil was performed using TRACE GC ULTRA (Thermo Fisher, USA). A constant flow of 1 mL min^−1^ of carrier gas (helium) was maintained throughout the whole analysis process. The injector temperature of the instrument was kept at 220 °C, while the oven temperature was programmed to start at 50 °C, (hold time 5.0 min) and ramped to 250 °C at a rate of 4 °C min^−1^ (hold time 5 min). A 1 μL sample (injection volume) was injected using a split mode (1:50) method. The ion source temperature was set at 220 °C, and the transfer line temperature was set at 270 °C. The electron impact mode was used for sample ionization (voltage of −70 eV), and the mass range varied from m/z 50 to 650 amu. Identification of individual compounds was carried out by comparing their mass spectra with those in the internal reference mass spectra library (NIST/Wiley). GC–MS analysis of the samples was performed in triplicate. The relative number of individual components was based on the peak area obtained from the detector response.

#### 2.3.5 Molecular docking and simulation studies

##### 2.3.5.1 *In silico* studies

The inhibitory action of the main antimicrobial agent—EC oil— in the formulated anti-dandruff hair oil against *M. furfur* was further assessed through *in silico* studies using AutoDock Vina 1.5.7,^1^ targeting lanosterol 14α-demethylase (LDM), an enzyme playing a crucial role in the biosynthesis of the fungal cell membrane. The 3D structure of LDM with co-crystallized ligand fluconazole (PDB ID: 4WMZ) was obtained from the RCSB database.^2^ To prepare the enzyme for docking, the co-crystallized ligand from the enzyme was initially removed and further optimized for gaps and charge filling using the SPDBv tool.^3^ The chemical structures of all quantified potent phytomolecules in EC oil were obtained from the PubChem database.^4^ For docking, the ligands and the protein file were optimized and converted into PDBQT format using AutoDock Tools 1.5.7 (Trott and Olson, 2010). The catalytic site of the enzyme was analyzed by visualizing the docking site of the co-crystallized ligand. Subsequently, a box enclosing the centroids of the co-crystallized ligand was defined as a grid with dimensions 22x40x54Å. Visualization of enzyme–ligand interactions and their subsequent analysis were performed using LIGPLOT+ v1.4.5 (Wallace et al., 1995; Laskowski and Swindells, 2011) and PyMOL (PyMOL Molecular Graphics System, Version 2.0, Schrodinger, LLC.sx).

##### 2.3.5.2 Molecular dynamics simulation

Lead compounds obtained from the docking studies were subjected to molecular dynamics (MD) simulations to evaluate the effects of ligand binding on the protein and the stability of the ligand within the binding site of the protein. The simulations were carried out for the independent protein and three ligand-bound protein complexes using GROMACS (Version: 2020.1) (Abraham et al., 2015; Abraham et al., 2020) with the Charmm27 force field. Topologies and parameter files of the lead compounds were generated using the SwissParam server (Zoete et al., 2011). The protein and the complexes were solvated using the single-point charge (SPC) water model extended approximately 10 nm in a cubic periodic box. For the electro-neutralization of the systems, the respective numbers of sodium and chloride ions were added. Energy minimization was performed using 50,000 steps of the steepest descent method to resolve any bad contacts and clashes in the protein. Following energy minimization, all systems underwent two steps of equilibration: first, 100 ps of canonical (NVT) equilibration, followed by 100 ps of isobaric-isothermic (NPT) equilibration. The temperature of the system was maintained at 300 °C using a modified Berendsen thermostat, and the pressure was maintained at 1 bar using the Berendsen barostat (Berendsen et al., 1984). MD simulations were performed for 5 ns, and the coordinates were saved every 10 ps for all systems. The resulting trajectories were visualized using the PyMol software (The PyMOL Molecular Graphics System, Version 2.0 Schrödinger, LLC.sx), and graphical representations were generated using the Grace software (Version 5.1.22).^5^

#### 2.3.6 Cell imaging by confocal microscopy

A rapid, double-staining procedure using fluorescein diacetate (FDA) and propidium iodide (PI) was applied to confirm the antimicrobial activity of the developed formulation through the determination of cell viability in cell suspensions (Zhu et al., 2023). Treated cells of dandruff-causing organisms were harvested from different treatments and incubated with a mixture of FDA (2 mg/mL) and PI (1 mg/mL). The stained cells were washed aseptically using PBS (pH 7.0). The viability of live and dead cells after the supplementation of the formulated product was visualized using a confocal laser scanning microscope and compared with respective controls and standards (Carl Zeiss LSM 510 META) using FDA (green) and PI (red) filters with excitation and emission at 480/40 nm and 630/30 nm, respectively. Furthermore, the fluorescence intensities of FDA and PI were individually extracted from replicate micrographs using the ZEISS ZEN software integrated with the confocal microscope and quantitatively analyzed to assess live and dead cell populations.

#### 2.3.7 Scanning electron microscopy

The antagonistic effects of the most effective final formulation against *M. furfur* cells were visualized using scanning electron microscopy (SEM), following the procedure outlined by Mishra et al. (2018). Treated *M. furfur* cells (supplemented with formulated herbal oil) were harvested by centrifugation at 10000 rpm for 5 min. Subsequently, the treated cells were fixed with 2.5% (v/v) glutaraldehyde containing 0.1 M sodium cacodylate buffer, followed by the treatment of OsO_4_ (1%w/v) for 40 min. Thereafter, dehydration of the treated cells was achieved using increasing concentrations of ethanol (30–100%). The cells were dried using a critical point dryer and coated with gold–palladium using a sputter coater (Q150T ES, Quorum, East Sussex, UK). The treated cells were then visualized using a scanning electron microscope (eSEM-FEI Quanta 250). All experimental procedures were performed in triplicate.

### 2.4 Statistical analysis

All experiments were performed in three biological replicates, and the observations were recorded as mean ± standard error (SE). The results were analyzed using the SPSS version 18.0 software with advanced models (SPSS Japan, Tokyo, Japan). Differences between means were analyzed using Tukey’s multiple comparison test (*p* ≥ 0.05). The remaining statistical observations were examined using GraphPad Prism (GraphPad Software Inc. 9.3.1, San Diego, CA, USA).

## 3 Results

### 3.1 Pre-formulation studies

#### 3.1.1 Inhibitory effect of the selected herbs against dandruff-causing agents

**Figure 1:**
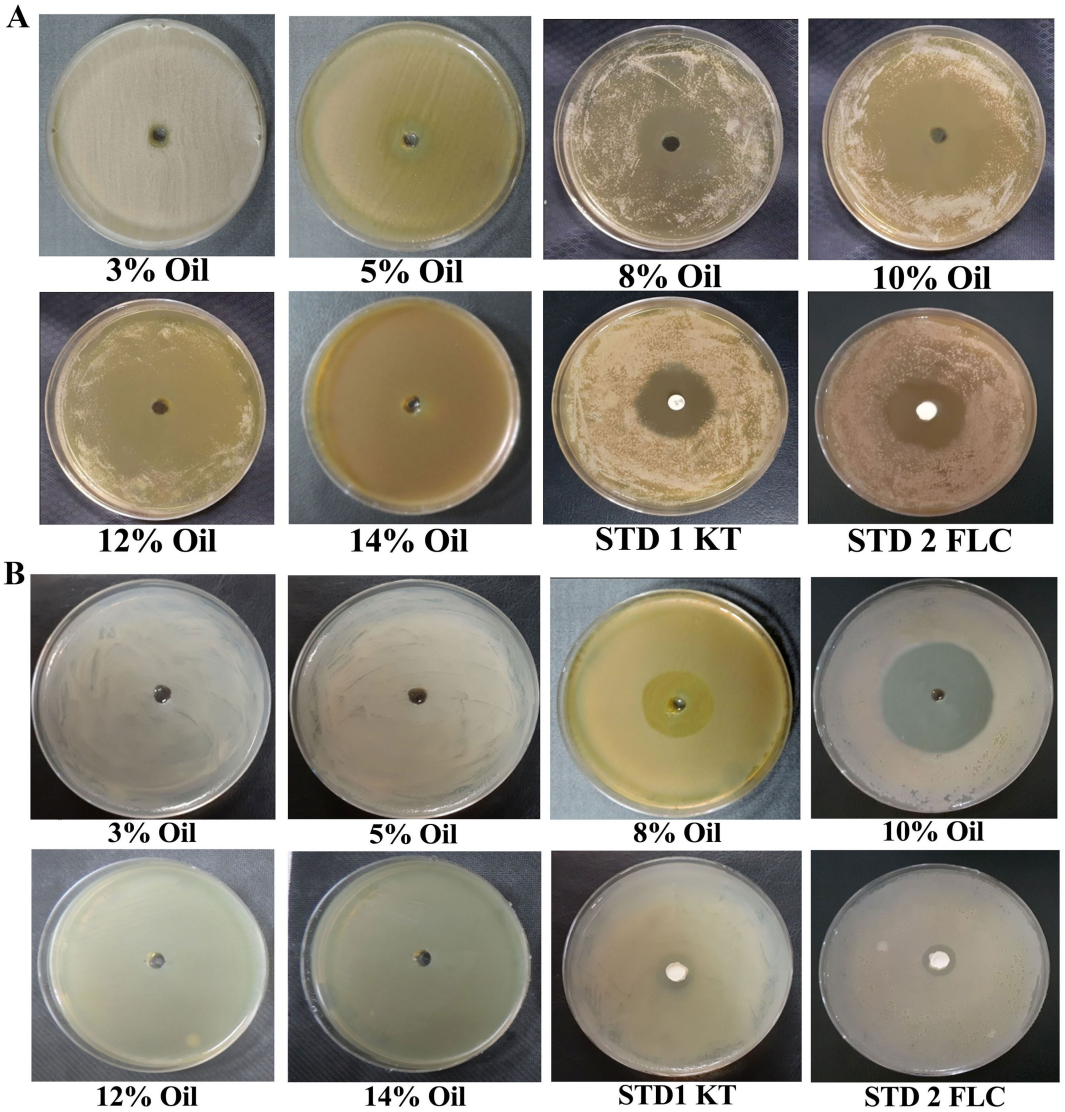
Antimicrobial activity of *E. citriodora* oil against dandruff-causing **(A)** *M. furfur*, and **(B)** *S. epidermidis.*

The inhibitory effect of EC oil (at different concentrations) was investigated against dandruff-causing fungal (*M. furfur*) and bacterial (*S. epidermidis*) inhabitants using the agar well diffusion assay. The findings revealed that EC oil showed very promising antimicrobial activity against these dandruff-causing pathogens, and the effect gradually increased with concentration (Figures 1A,B). Notably, the highest inhibition zone (37.3 ± 1.8 mm) was observed at 12% concentration and showed a 1.32- and 1.39-fold increase compared to standard 1 (STD1; ketoconazole 50 mg) and standard 2 (STD2; fluconazole 50 mg), respectively (Supplementary Table S5.1). Similarly, a 1.27- and 1.35-fold increase in antimicrobial activity was observed at 10% concentration of EC oil (36 ± 2.08 mm inhibition zone) compared to STD1 and STD2 against *M. furfur*. In contrast, 8% EC oil displayed the least inhibitory effect against *M. furfur.* Noticeably, no statistically significant difference was observed in the inhibitory effect of 12 and 10% concentrations of EC oil against *M. furfur* (Figure 1A; Supplementary Table S5.1). Whereas, 10% concentration of EC oil was depicted maximum inhibitory activity against *S. epidermidis* (38.7 ± 0.9mm), estimated 2.8 and 2.76-fold increment (Figure 1B; Supplementary Table S5.2). Moreover, 8% EC oil also exhibited a considerable inhibition zone (24 ± 0.9 mm) against *S. epidermidis*, which was 1.76- and 1.71-fold higher than the respective standards (Supplementary Table S5.2). The antimicrobial activity of *C. asiatica* and *W. trilobata* was not as pronounced (Supplementary Table S4). However, these herbs are traditionally known for their various other properties such as wound healing and maintaining the luster and texture of hair strands (Bylka et al., 2013; Arribas-López et al., 2022).

#### 3.1.2 Herbal ingredient-mediated formulation development

Based on the physiological and physicochemical characteristics, along with the IC_50_ value of the selected herbs against dandruff-causing pathogens, different permutation combinations (*n* = 3) were prepared to identify a novel and effective herbal formulation. The permutation combinations of synergistic herbal formulation comprised the pharmaceutically acceptable concentrations of *Centella asiatica* (3.0–6.0 w/v), *Wedelia trilobata* (2.0–5.0 w/v), and EC oil (8–12% v/v).

### 3.2 Post-formulation studies

#### 3.2.1 Anti-dandruff activity of the formulated hair oil

The formulated combinations were tested against major dandruff-causing microbes using the agar well diffusion assay to evaluate their synergistic antimicrobial activity compared to standards and market products (MP1 and MP2). The observations indicated that the formulated combinations exhibited higher inhibitory activity against *M. furfur* (Table 1) and *S. epidermidis* (Table 2) compared to the individual herbal ingredients.

**Table 1:** Antimicrobial activity of herbal constituents (at different concentrations) against *M. furfur* and their comparison with the permutations and combinations of the developed formulations.

Selected samples	Developed formulation	*E. citriodora* essential oil		*Centella asiatica*		*Wedelia* spp.	
	Inhibition (mm)	Concentrations	Inhibition (mm)	Concentrations	Inhibition (mm)	Concentrations	Inhibition (mm)
Control	0 ± 0.0	Control	0 ± 0.0	Control	0 ± 0.0	Control	0 ± 0.0
*Formulation 1* (F1)	25.3 ± 1.5^c^	8% oil	20.33 ± 0.0^c^	20 mg/mL	9.0 ± 0.0^c^	20 mg/ml	9.33 ± 0.57^b^
*Formulation 2* (F2)	39.3 ± 1.5^a^	10% oil	36 ± 2.08^a^	40 mg/mL	12.3 ± 0.57^b^	40 mg/ml	11 ± 1.0^b^
*Formulation 3* (F3)	41.3 ± 1.5^a^	12% oil	37.3 ± 1.18^a^	60 mg/mL	12.7 ± 0.6^b^	60 mg/ml	10.33 ± 0.6^b^
Market Product 1	13.7 ± 0.58^e^	–	–	MP1	–	MP1	–
Market Product 2	16.7 ± 1.5^d^	–	–	MP2	–	MP2	–
STD1 KT	28.3 ± 1.5^b^	STD1 KT	28.3 ± 1.5^b^	STD1 KT	28.3 ± 1.5^a^	STD1 KT	28.3 ± 1.5^a^
STD2 FLC	26.7 ± 1.5^c^	STD2 FLC	26.7 ± 1.5^bc^	STD2 FLC	26.7 ± 1.5^a^	STD2 FLC	26.7 ± 1.5^a^

*Inhibition zone (in mm) values are the mean of three independent replicates with ± standard error. **Means followed by the same letter(s) within the column are not significantly different according to Tukey’s multiple comparison test (*p* < 0.05).

**Table 2:** Antimicrobial activity of herbal constituents (at different concentrations) against *S. epidermidis* and their comparison with the permutations and combinations of the developed formulations.

Selected samples	Developed formulation	*E. citriodora* essential oil		*Centella asiatica*		*Wedelia* spp.	
	Inhibition (mm)	Concentrations	Inhibition (mm)	Concentrations	Inhibition (mm)	Concentrations	Inhibition (mm)
Control	0 ± 0.0	Control	0 ± 0.0	Control	0 ± 0.0	Control	0 ± 0.0
*Formulation 1* (F1)	27.3 ± 0.58^b^	8% oil	24 ± 2.64^b^	20 mg/ml	10.67 ± 0.58^b^	20 mg/ml	11 ± 1^b^
*Formulation 2* (F2)	41.7 ± 1.5^a^	10% oil	38.7 ± 1.52^a^	40 mg/ml	12.3 ± 1.5^ab^	40 mg/ml	12 ± 1.0^b^
*Formulation 3* (F3)	NG	12% oil	NG	60 mg/ml	13 ± 1^ab^	60 mg/ml	11.6 ± 0.6^b^
Market Product 1	14.7 ± 0.58^d^	–	–	MP1	–	MP1	–
Market Product 2	20.3 ± 1.5^c^	–	–	MP2	–	MP2	–
STD1 KT	12.7 ± 1.15^e^	STD1 KT	13.6 ± 0.33^c^	STD1 KT	13.6 ± 0.33^a^	STD1 KT	13.6 ± 0.33^a^
STD2 FLC	11.3 ± 0.58^e^	STD2 FLC	14.0 ± 0.6^c^	STD2 FLC	14.0 ± 0.6^a^	STD2 FLC	14.0 ± 0.6^a^

*Inhibition zone (in mm) values are the mean of three independent replicates with ± standard error. **Means followed by the same letter(s) within the column are not significantly different according to Tukey’s multiple comparison test (*p* < 0.05). ***NG, No Growth.

#### 3.2.2 Effect of the formulated oil on the average cell count of dandruff-causing agents

As evidenced by the agar well diffusion assay, the developed formulation diminished the growth and proliferation of dandruff-causing microbes. The results showed the lowest yeast cell viability at 10 and 12% concentrations of EC oil. Moreover, a 1.8- and 1.9-fold reduction in the average cell count of *M. furfur* was recorded in formulation 2 (10% EC oil) and 3 (12% EC oil), respectively, compared to their respective controls (Table 3). Furthermore, the reduction in *M. furfur* growth observed with formulations 2 and 3 was significantly higher than that with STD1 (1.4- and 1.5-fold), STD2 (1.3- and 1.4-fold), MP1 (1.73- and 1.84-fold), and MP2 (1.68- and 1.80-fold), respectively. Among all developed permutations and combinations, formulation 1 (8% EC oil) exhibited the least inhibitory activity against *M. furfur*.

**Table 3:** Effect of the developed formulations on the average spore count of *M. furfur* and the average cell count (log CFU) of *S. epidermidis.*

Treatments	Average yeast count (log spore count)	Average bacterial count (log CFU count)
Control	5.61 ± 0.05^a^	5.7 ± 1.2^a^
Formulation 1 (F1)	5.12 ± 0.12^b^	3.52 ± 0.5^d^
Formulation 2 (F2)	3 ± 0.06^d^	2.8 ± 0.02^f^
Formulation 3 (F3)	2.8 ± 0.23^d^	2.5 ± 1.2^f^
Market Product 1	5.1 ± 0.5^b^	4.8 ± 1.5^b^
Market Product 2	5.06 ± 0.7^b^	4.1 ± 0.4^c^
Standard 1 (KT 50 mg)	4.36 ± 1.2^c^	4.36 ± 0.2^c^
Standard 2 (FLC 50 mg)	4 ± 0.83^c^	4.98 ± 0.35^b^

Means followed by the same letter(s) within the column are not significantly different according to Tukey’s multiple comparison test (*p* < 0.05).

Similarly, the reduced average CFU count of *S. epidermidis* following treatment with formulations 1, 2, and 3 further demonstrates their inhibitory effect. The results, showing the least average CFU count of *S. epidermidis* in the formulated hair oil treatment, suggest that formulations 2 and 3 were the most effective combination for inhibiting the growth of *S. epidermidis* (2- and 2.5-fold) compared to the control and recorded 1.76- and 1.97-fold greater reductions than STD2 (Table 3).

#### 3.2.3 Formulated oil diminishes the cell viability of dandruff-causing agents

**Figure 2:**
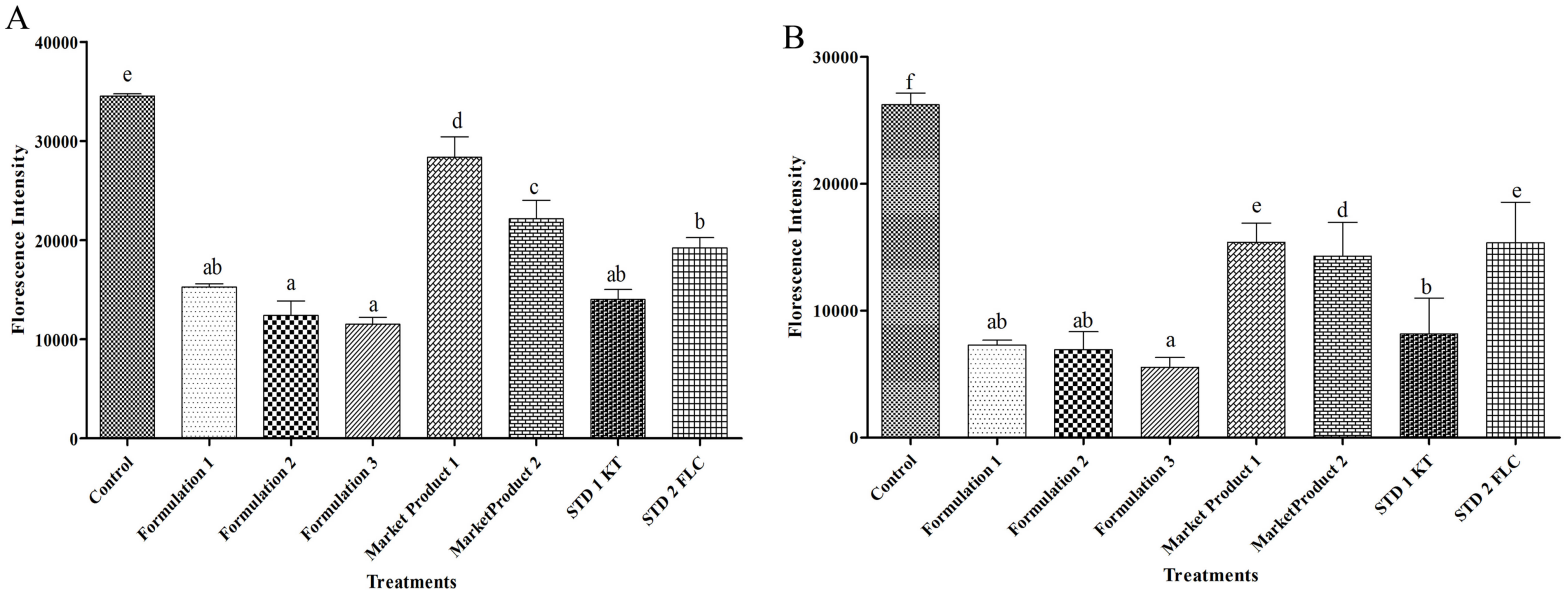
FDA-based cell viability of *M. furfur* **(A)** and *S. epidermidis* **(B)** after treatment with *E. citriodora* oil. Means followed by the same letter(s) within the column are not significantly different according to Tukey’s multiple comparison test (*p* < 0.05).

The intensity of FDA fluorescence was comparatively low when the cells were treated with the formulated oil. Moreover, the lowest fluorophore uptake (3- and 2.8-fold compared to the control) in the *M. furfur* cells was detected after treatment with formulations 3 and 2. These reductions were greater than those observed with MP1 (2.46- and 2.28-fold), MP2 (1.92- and 1.78-fold), STD1 (1.67- and 1.54-fold), and STD2 (1.21- and 1.13-fold). Formulation 1 resulted in higher FDA fluorescence (1.3 and 1.2-fold) in the *M. furfur* cells compared to formulations 3 and 2, although it still showed a 2.26-fold reduction compared to the respective control (Figure 2A).

Treatment with the formulated herbal anti-dandruff hair oil (in different permutation combinations) was most effective against *S. epidermidis* and recorded 3.59-, 3.78-, and 4.72-fold reductions in the intensity of FDA compared to their respective control (Figure 2B). Among these, treatment with formulations 3 and 2 resulted in the most significant reduction in fluorophore uptake by *S. epidermidis* cells, compared to MP1 (2.77- and 2.22-fold), MP2 (2.57- and 2.06-fold), STD1 (1.47- and 1.18-fold), and STD2 (2.8- and 2.2-fold), respectively. Although formulation 1 exhibited a 2.1-, 1.95-, 1.1-, and 2.1-fold greater reduction in FDA intensity compared to MP1, MP2, STD1, and STD2, respectively, this reduction was still lower than that observed in other permutations and combinations of the formulated herbal anti-dandruff hair oil (Figure 2B).

#### 3.2.4 Quality regulation of the selected herbal leads

*C. asiatica*, *W. trilobata,* and EC oil were subjected to physicochemical analysis to ascertain the identity, purity, and strength of the raw material used in the study (Supplementary Tables S1, S2). Data suggest that the quality of the raw material complied with the standards of the Ayurvedic Pharmacopoeia of India.

#### 3.2.5 Chromatographic profiling of the selected herbal leads

HPLC profiling confirmed the presence of bioactive markers, namely MS (3.69 μg/mg), AS (1.87 μg/mg), and MA (1.084 μg/mg), with retention times (R_t_) of 4.121 min, 5.017 min, and 7.667 min, respectively, in the *C. asiatica* extract, while AA could not be detected due to its very low content (Supplementary Figure S1). Similarly, WDL (32.6 μg/mg) was detected at 6.273 min in the *W. trilobata* extract (Supplementary Figure S1). The EC oil was found to be rich in isopulegol (42.26%), followed by citronellol (8.54%), p-Methane-3-one (4.79%), and citronella formate (3.64%). I-Hepta triacontanol was found in the least concentration, i.e., 0.87% (Supplementary Figure S2; Supplementary Table S3).

#### 3.2.6 Molecular docking and MD simulation studies

**Figure 3:**
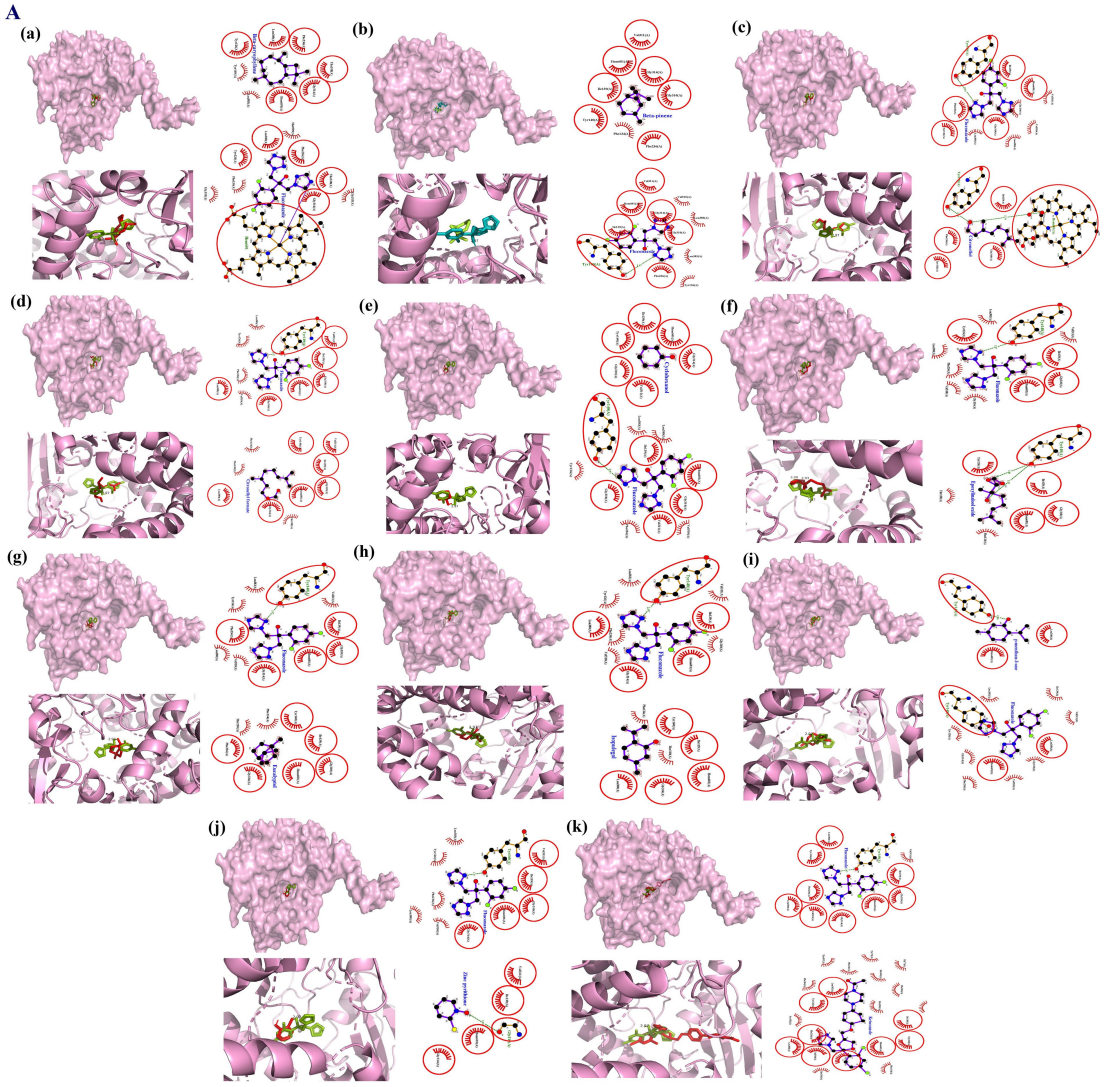
Molecular interactions between lansterol-14α-demethylase (a component of the *M. furfur* cell wall; PDB ID: 4WMZ) and phytomolecules from EC oil, investigated by molecular docking. The figures depict the surface view of the ligand-binding site overlaid with the co-crystalized ligand and highlight their shared interactions within red circles. In this figure **(a)** *β*-caryophyllene; **(b)** β-pinene; **(c)** Citronellol; **(d)** Citronellyl formate; **(e)** Cyclohexanol; **(f)** Epoxylinalool oxide; **(g)** Eucalyptol; **(h)** Isopulegol; **(i)** p-menthan-3-one; **(j)** Zinc pyrithione; **(k)** Ketoconazole.

Molecular docking analysis of EC oil revealed the binding of all phytomolecules at the inhibition site of LDM with different affinities (Supplementary Table S6). The binding at the inhibition site was confirmed by visualizing the overlapping interactions between the co-crystallized ligand and the phytomolecules, which showed hydrophobic contacts with His601, Thr318, Phe236, Leu380, Gly314, Ile139, and Gly310, along with hydrogen bonding to Tyr140 (Figure 3). Among the quantified phytomolecules of EC oil, *β*-caryophyllene showed the highest docking score (∆G = −8.9 Kcal/Mol), followed by eucalyptol (∆G = −6.8 Kcal/Mol) and isopulegol (∆G = −6.5), compared to respective standards—ketoconazole (∆G = −10.3) and fluconazole (∆G = −8.2).

**Figure 4:**
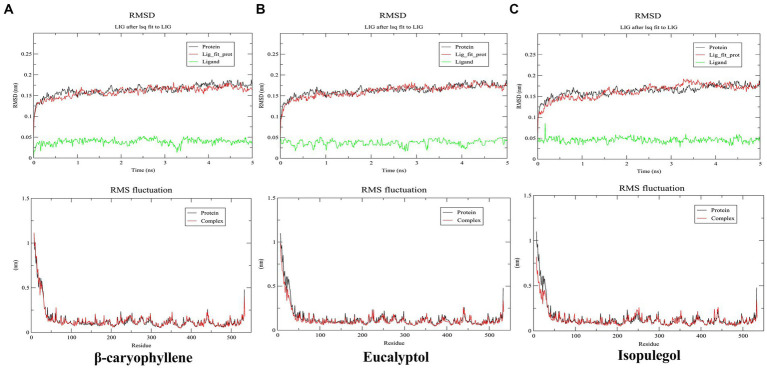
Root mean square deviation (RMSD) and root mean square fluctuation (RMSF) of β-caryophyllene **(A)**, eucalyptol **(B)**, and isopulegol **(C)**.

As per the docking results, β-caryophyllene, eucalyptol, and isopulegol were selected for MD simulation to examine the dynamic behavioral effects of their binding on the target protein. The stability of the protein and the protein–ligand complex during the 5 ns simulation was monitored using the time evolution of the root mean square deviation (RMSD), as shown in Figure 4. The RMSD of all complexes and the unbound protein remained >0.20 nm, indicating the stability of the systems. In the β-caryophyllene–bound complex (A), the final RMSD was slightly lower than that of the protein, suggesting increased rigidity of the protein upon ligand binding. However, in both the eucalyptol (B) and isopulegol complexes (C), no significant deviation in RMSD was observed compared to the unbound protein, indicating no significant alteration in protein stability due to ligand binding. The root mean square fluctuation (RMSF) of the residues was compared to assess the mobility of the residues in the protein and protein–ligand complex systems, as influenced by ligand binding. The RMSF of all the complexes exhibited no significant fluctuation compared to the unbound protein, indicating that ligand binding does not disrupt or restrict the motion of residues. RMSF values in all systems ranged below 0.25 nm throughout the binding pocket (except for the terminals of the protein), suggesting the stability of the active site after ligand binding.

#### 3.2.7 Localization of the live/dead cells of *M. furfur* after treatment with the formulated oil

**Figure 5:**
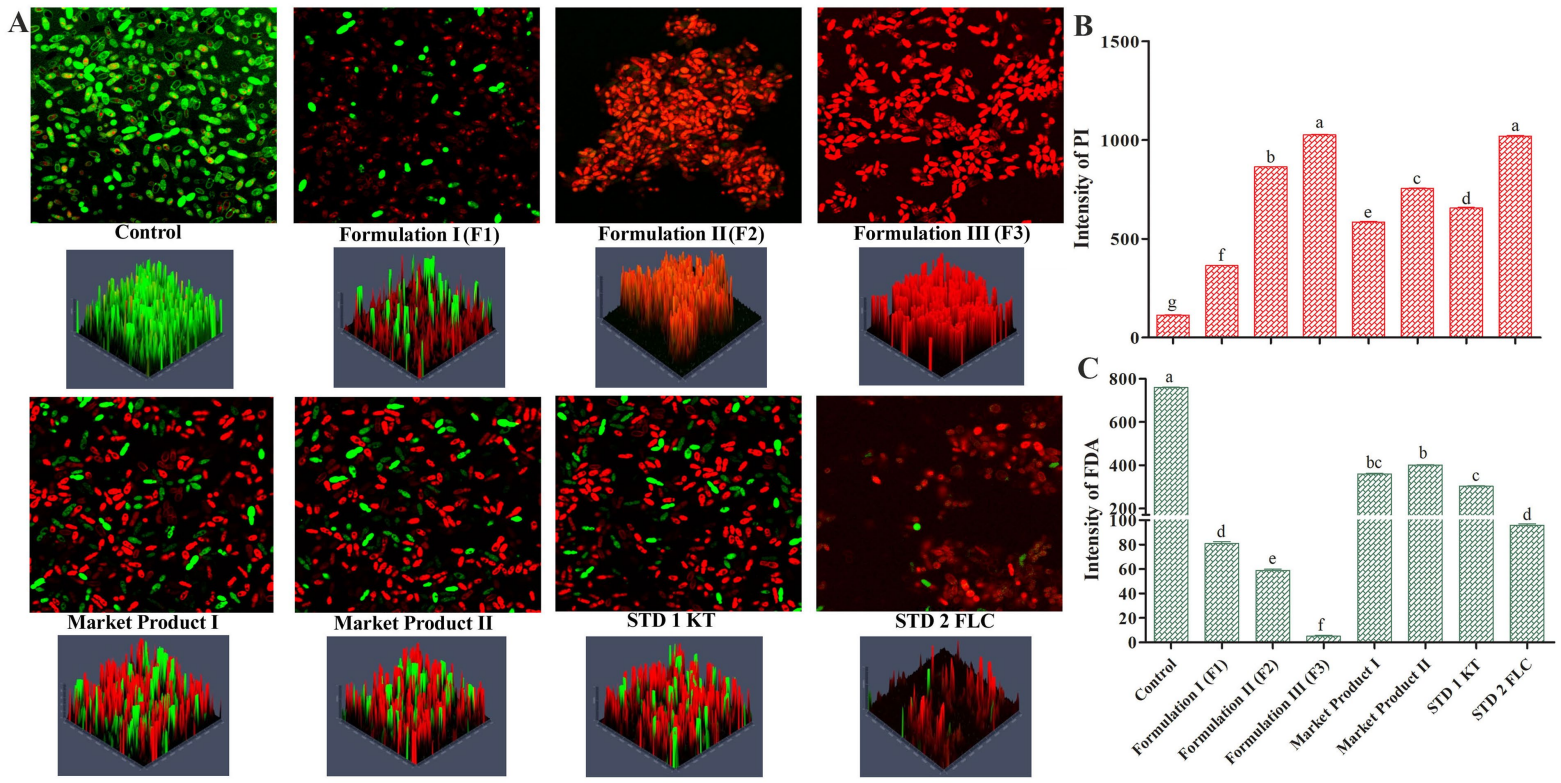
**(A)** Confocal microscopy-based localization of M. furfur live/dead cells treated with different permutations and combinations of the formulated oil. The treated cells were co-stained with propidium iodide (PI) and fluorescein diacetate (FDA). Average fluorescence intensity of PI **(B)** and FDA **(C)** in M. furfur spores after treatments. Means followed by the same letter(s) within the column are not significantly different according to Tukey’s multiple comparison test (*p* < 0.05).

The findings of molecular docking were validated *via* live/dead cell imaging by confocal microscopy. The localization of live/dead cells under different treatments clearly depicted that formulations 3 and 2 (containing 12 and 10% EC oil) were the most effective combinations compared to market products, standards, and the respective control. Detailed analysis of confocal micrographs showed the highest number of live cells in the untreated microbial population (Figure 5A), whereas treatments with MP1, MP2, STD1, and STD2 resulted in a reduction of live cells. The fewest live cells were observed in the formulated oil treatments (in various permutation combinations), as evidenced by the strongest PI fluorescence under the same conditions. Moreover, the findings of confocal microscopy were corroborated by the average intensity ratios of PI (Figure 5B) and FDA (Figure 5C) fluorescence.

It is noteworthy that formulations 2 and 3 did not show significant variation in terms of antimicrobial activity against dandruff-causing microbes, which is consistent with the findings of previous experiments. Therefore, formulation 2 (containing 10% EC oil) was selected as the best-suited anti-dandruff hair oil.

#### 3.2.8 Scanning electron microscopy of *M. furfur* cells treated with the formulated oil

**Figure 6:**
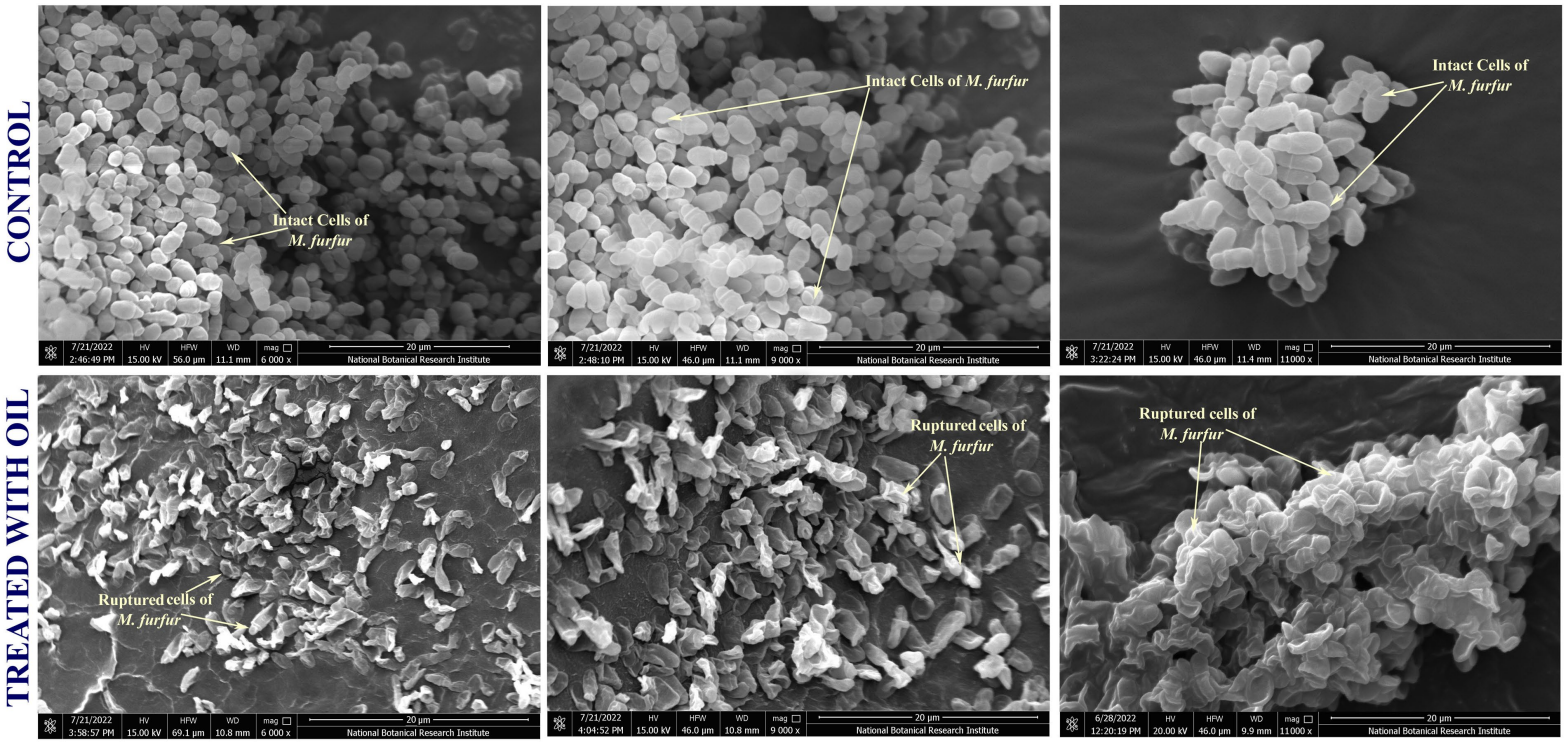
Scanning electron microscopy (SEM) images of *M. furfur* cells after treatment with the final formulated anti-dandruff oil.

Scanning electron microscopy (SEM) was performed to evaluate the effect of the final formulation of the anti-dandruff herbal hair oil against *M. furfur.* The SEM micrographs clearly showed punctured *M. furfur* cells with rough, disintegrated, and degraded surfaces after exposure to the final formulation (Figure 6). In contrast, untreated control *M. furfur* cells appeared smooth and intact (Figure 6).

## 4 Discussion

Dandruff-induced hair fall is a serious dermatological issue, affecting half of the global population (Elewski, 2005). At some point in their lives, everyone experiences an infection from dandruff-causing organisms. Among them, *M. furfur* and *S. epidermidis* are the most common and very devastating pathogens, causing pityriasis versicolor, dandruff, and seborrheic dermatitis in humans (Gupta et al., 2004; Clavaud et al., 2013). A physiological study of scaling estimated that approximately 800,000/sq. cm cells get released from the compromised scalp at once after infection by these dandruff-causing microbes (Arndt and Hsu, 2007). Considerable efforts have been made so far to suppress the pathogenicity of dandruff-causing organisms. These efforts include the application of several synthetic formulations based on ketoconazole, fluconazole, and itraconazole (Leong et al., 2021; Godge et al., 2023); chemical polypeptides such as zinc pyrithione (Reeder et al., 2011; Mangion et al., 2023); salicylic acid (Massiot et al., 2023); tar and steroids (Franchimont et al., 2003; Sawleshwarkar et al., 2004); and other compounds such as propylene glycol and selenium sulfide (Back, 2009; Saunte et al., 2020). Unfortunately, the use of these formulations is restricted because they fail to cure or prevent the recurrence of dandruff, lead to the development of resistance in dandruff-causing pathogens, and can cause certain kinds of highly toxic and adverse effects on the scalp (Trüeb, 2007; Okokon et al., 2015; Chowdhry et al., 2017; Kubicki et al., 2020; Lopes et al., 2024). Therefore, there is an unmet need for alternatives to these synthetic formulations that do not have any negative impact on the human scalp and are able to eradicate dandruff completely. Moreover, it could also be possible that the solution lies not in synthetic formulations but within the realm of nature itself.

Ayurveda offers a potential solution to overcome these issues, employing selected herbs in a synergistic manner to treat and prevent infections caused by dandruff-causing microorganisms. Moreover, an oil-based, scientifically validated herbal formulation for the treatment of dandruff has not been extensively explored yet. For this reason, the present study aimed to investigate the efficacy of plant-based ingredients against dandruff-causing organisms and develop a synergistically active and scientifically validated anti-dandruff hair oil formulation.

Several researchers have emphasized the antimicrobial properties of *Eucalyptus* essential oil (Batish et al., 2008), reporting that the essential oil of *Eucalyptus* has immense potential as an antibacterial (Cimanga et al., 2002) and antifungal (Ruchi Tiwari et al., 2017) agent. In addition, a few studies have also shown the anti-dermatophytic activity of *Eucalyptus* oil against *Microsporum canis, Microsporum gypseum, Trichophyton mentagrophytes,* and *Trichophyton rubrum* (Tolba et al., 2015). Furthermore, various species of *Eucalyptus,* such as *Eucalyptus camaldulensis*, *Eucalyptus globulus*, *Eucalyptus maculate,* and *Eucalyptus viminalis,* were tested against *Trichophyton mentagrophytes* (Takahashi et al., 2004). However, the anti-dandruff activity of EC oil against *M. furfur* and *S. epidermidis* remains unexplored. In the present study, the findings of the agar well diffusion assay clearly demonstrated that EC oil efficiently suppressed the growth of the main causative agents of dandruff—*M. furfur* and *S. epidermidis—*at different concentrations (Figures 1A,B; Tables 1, 2).

Despite a few studies on the antibacterial activity of *Wedelia* spp. (Darah et al., 2013), *C. asiatica* and *W. trilobata* have not been extensively studied for their anti-dandruff properties. The observations of the current study align with earlier findings, as they did not show any inhibitory effects against dandruff-causing microbes (Supplementary Tables S4.1–S4.4). However, these plants (*C. asiatica* and *W. trilobata*) were used for their therapeutic potential, such as improving hair luster, promoting wound healing, and others. Their wide range of pharmacological activities supports their use in formulation development. *In vitro* and *in vivo* studies have demonstrated that centellosides of *C. asiatica* positively induce signaling pathways, exerting therapeutic effects on various skin conditions (Bylka et al., 2014; Park, 2021), as well as psoriasis and scleroderma (Sun et al., 2020). Moreover, it also enhances the proliferation of fibroblasts, elevates collagen and intracellular fibronectin content, ameliorates the tensile strength of young skin, and reduces hypertrophic scars and keloids (Bylka et al., 2013; Arribas-López et al., 2022). In contrast, *W. trilobata* has been frequently used as an ingredient in different formulations for treating hair fall and skin diseases (Meena et al., 2011) (Patent no. JP3542700B2; JP6977233B2; US20090104295A1; US7910557). Similarly, it has been used in Ayurveda to remove scalp scurf, dye hair, cure alopecia, and promote hair growth (Dutt, 1877; Mohan et al., 2021). Therefore, anti-dandruff oil supplemented with these herbal extracts not only eliminates dandruff but also nourishes the scalp and promotes hair luster and growth, preventing the recurrence of dandruff-causing organisms.

Furthermore, different permutation combinations of herbal formulations were developed based on the anti-dandruff activity of EC oil and the therapeutic properties of *C. asiatica* and *W. trilobata*. These combinations were examined for their synergistic effect on dandruff-causing organisms, *M. furfur* and *S. epidermidis*, to assess the most effective combination. Similar to the agar well diffusion assay, the highest reduction in *M. furfur* and *S. epidermidis* cells/spores was recorded after treatment with these combinations (Table 3). The reduction in the viable cell count could be because of the synergistic anti-dandruff properties of the selected herbs, which led to the development of effective formulations (Chaiyana et al., 2022). The present study’s findings are consistent with earlier reports, where the extracts of *Erythrina caffra* Thunb. (Olajuyigbe and Afolayan, 2012) and *Herniaria glabra* (Wojnicz et al., 2012) effectively reduced the viable cell count of bacterial pathogens.

Gas chromatography profiling confirmed the presence of several key components in EC oil, including 1,8-cineole (Eucalyptol), *β*-pinene, citronellol, and β-caryophyllene. This finding aligns with previous reports (Sebei et al., 2015; Tolba et al., 2015; Luís et al., 2016). Moreover, these components have been found to show anti-oxidant and anti-inflammatory effects and are considered safe for topical application, with no reported cases of skin irritation (Ramezani et al., 2002; Ramsey et al., 2020; Sindle and Martin, 2021). Molecular docking of these compounds showed higher binding affinity of β-caryophyllene, eucalyptol, and isopulegol at the binding sites of lanosterol 14α-demethylase (an enzyme responsible for the synthesis of the cell membrane in *M. furfur*), which leads to the malformation of the *M. furfur* cell membrane (Figure 3), thereby decreasing the risk of seborrheic dermatitis or dandruff (Thomas and Khasraghi, 2020). Despite lacking an azole ring, these phytomolecules were found to be comparable to ketoconazole and other synthetic compounds in terms of binding affinity at the inhibition sites. In addition, these phytomolecules were found to bind with heme iron, an important step in interrupting the fungal ergosterol biosynthetic pathway, similar to the mechanism of azole-derived antifungal drugs (Yang et al., 2015; Martinez-Rossi et al., 2018).

Since *M. furfur* infection also creates favorable conditions for *S. epidermidis* colonization by increasing pH and provides a lipid-free environment (Han et al., 2019), we investigated whether the formulated anti-dandruff oil could suppress *M. furfur* growth. FDA/PI-based confocal microscopy indicated that the cells of *M. furfur* treated with different permutation combinations showed the lowest level of FDA fluorescence in the confocal micrographs (Figure 5). Similarly, this was supported by an FDA-based fluorophore uptake experiment (Figure 2A) and the average fluorescence intensity of the corresponding micrographs (Figures 5B,C). The findings further confirm that the high mortality observed in the formulated anti-dandruff oil treatment is primarily attributed to the antimicrobial compounds present in Eucalyptus citriodora oil.

Furthermore, SEM micrographs of the treated *M. furfur* cells showed the potential of the formulated oil to cause cell wall disintegration (Figure 6). The punctured cells observed after treatment further validated the findings of molecular docking, which showed strong binding affinity of key phytomolecules to the heme iron moiety of LDM, suggesting the disruption of the ergosterol biosynthesis pathway as a plausible mechanism of action. These findings provide mechanistic insights; however, they warrant further validation through direct ergosterol quantification and gene expression analysis. Although the study provides comprehensive insights based on in vitro and in silico analyses, the lack of in vivo data remains a limitation that may influence the translational relevance of our findings. Future investigations involving in vivo assessments and clinical evaluations will be crucial to substantiate the efficacy, safety, and therapeutic potential of the developed formulation under real-world conditions.

## 5 Conclusion

The results of the present investigation suggest that *E. citriodora* oil possesses significant inhibitory effects against *M. furfur and S. epidermidis*, the main dandruff-causing organisms. In addition, the synergistic action of the ingredients in the formulated oil is evident when compared to the anti-dandruff potential of individual ingredient(s). The developed herbal anti-dandruff hair oil is effective in the management of dandruff by diminishing the pathogenicity of dandruff-causing agents and ameliorating the damage caused by them. Regular application of the formulated herbal anti-dandruff hair oil also limits the recurrence of dandruff.

## Data Availability

The original contributions presented in the study are included in the article/Supplementary material, further inquiries can be directed to the corresponding author.

## Footnotes

^1^ http://vina.scripps.edu/

^2^ https://www.rcsb.org/

^3^ https://spdbv.vital-it.ch/

^4^ www.pubchem.com

^5^ https://plasma-gate.weizmann.ac.il/Grace/
