# Supplementary material for: A synergistic herbal formulation targeting Malassezia furfur and Staphylococcus epidermidis for effective dandruff management
Source: Front Microbiol. 2025 Nov 21;16:1654658. doi: 10.3389/fmicb.2025.1654658 (PMC12679390; doi:10.3389/fmicb.2025.1654658)
Supplement: Supplementary file 1 [file Supplementary_file_1.docx]

**Supplementary 1**

***Experimental Materials:***

***1. Chemicals and Reagents***

The reference compounds Ketoconazole and Fluconazole were procured from Sigma Aldrich (St. Louis, MO, USA). The solvents and other chemicals used were of analytical grade. Marketed products (MP1 and MP2) were procured from local market.

***2. Procurement of reference biological material***

Pure microbial culture of *Malassezia furfur* (MTCC 1374) and *Staphylococcus epidermidis* (MTCC 435) were procured from National Biodiversity Authority (NBA) approved culture collection unit of Indian Institute of Microbial Technology (IMTECH, Chandigarh, India).

***3. Procurement of raw material***

The plant samples i.e. *Centella asiatica* and *Wedelia trilobata* were collected from the botanic garden of CSIR- National Botanical Research Institute (Lucknow), authenticated (by Dr. Sharad Srivastava, Senior Principal Scientist & Head, Pharmacognosy Division, CSIR-NBRI) and live specimen was deposited in the Raw Drug Repository (RDR) of institute. The *Eucalyptus citriodora* essential oil (>98% purity) was procured from M/s Shaivi Enterprises (Lucknow, Invoice No. 37).

***4. Extraction protocols***

The extraction was done following the method of ([Kang et al., 2011](#_ENREF_1)) with slight modifications. The collected plant samples were shade dried, followed by drying at 40°C in a hot air oven. Drying the plant material concentrates the target compounds per gram making extraction efficient, and more uniform due to better solvent penetration and better reproducibility ([Rocha et al., 2011](#_ENREF_3)). Fresh plants tissues contain several enzymes like oxidases, peroxidases, hydrolases etc. which are active while the plant is alive or recently harvested. These enzymes can degrade or modify bioactive compounds like phenolics, flavonoids, alkaloids etc. during the time between harvest and extraction. Drying reduces or stops these enzymatic activities by removing water and raising temperature enough to inactivate enzymes i.e., 40°C ([Krakowska-Sieprawska et al., 2022](#_ENREF_2)). The coarsely powdered samples (5g) was individually subjected to cold extraction in methanol, shacked continually for 6h and left to stand for 18h. The sample was filtered (Whatmann No. 4) and the residue was soaked again in fresh solvent. The extraction procedure was repeated thrice and the pooled filtrate was vacuum concentrated in a rotatory evaporator (Buchi Rotavpour, Switzerland) at 40°C. The concentrated extract was finally freeze-dried, yield calculated, and stored at 4°C for further use.

Kang, C.-G., Hah, D.-S., Kim, C.-H., Kim, Y.-H., Kim, E., and Kim, J.-S. (2011). Evaluation of antimicrobial activity of the methanol extracts from 8 traditional medicinal plants. *Toxicological research* 27(1)**,** 31-36.

Krakowska-Sieprawska, A., Kiełbasa, A., Rafińska, K., Ligor, M., and Buszewski, B. (2022). Modern methods of pre-treatment of plant material for the extraction of bioactive compounds. *Molecules* 27(3)**,** 730.

Rocha, R., Melo, E.C., and Radünz, L. (2011). Influence of drying process on the quality of medicinal plants: A review. *Journal of Medicinal Plants Research* 5(33)**,** 7076-7084.
