# Supplementary material for: A synergistic herbal formulation targeting Malassezia furfur and Staphylococcus epidermidis for effective dandruff management
Source: Front Microbiol. 2025 Nov 21;16:1654658. doi: 10.3389/fmicb.2025.1654658 (PMC12679390; doi:10.3389/fmicb.2025.1654658)
Supplement: Supplementary file 2 [file Supplementary_file_2.docx]

**
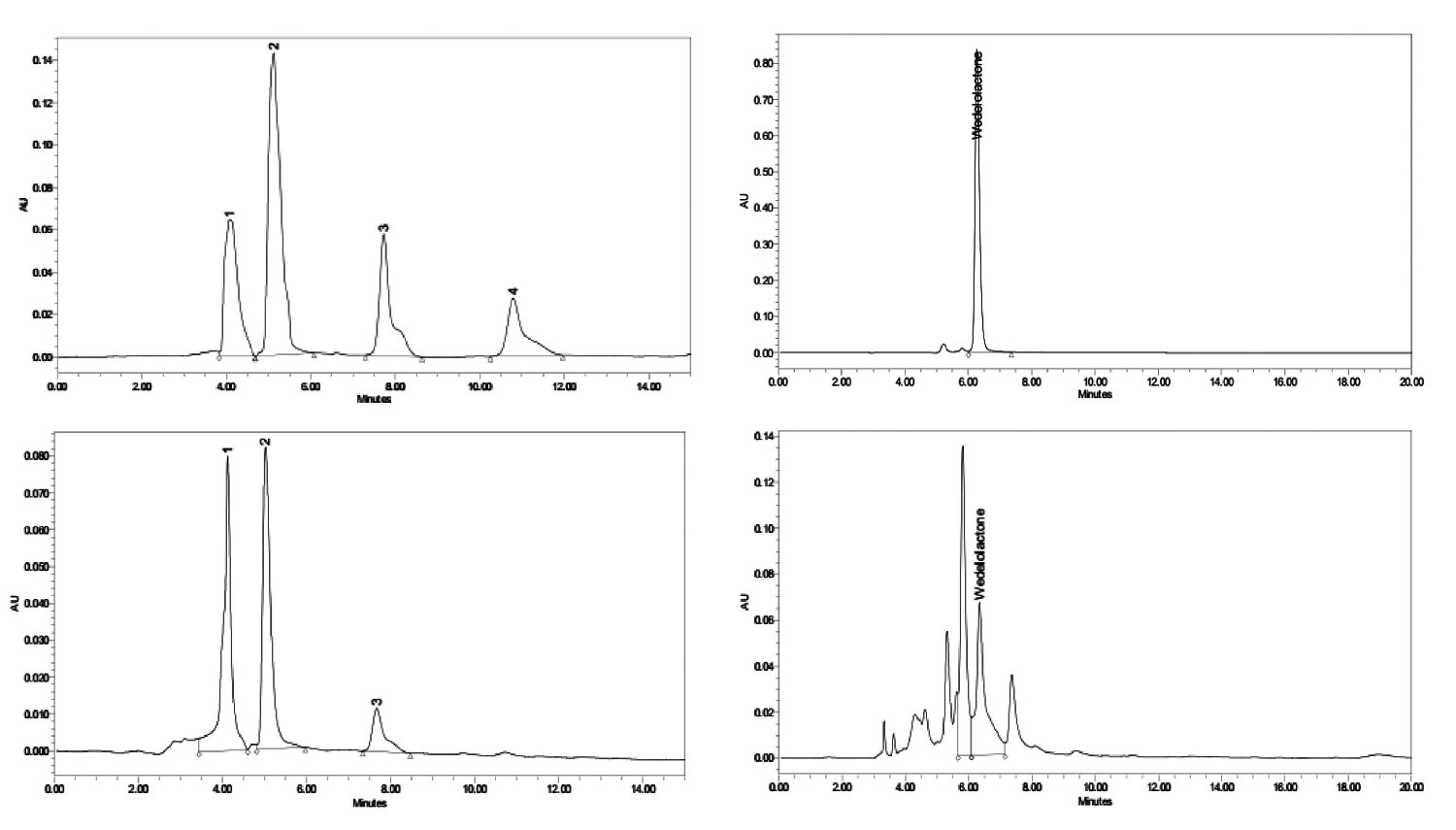
Supplementary Figure 1**

**Figure S1.** HPLC chromatogram of (A) mixture of *Centella asiatica* markers: peak 1- madecassoside, peak 2- asiaticoside, peak 3- madecassic acid, peak 4- asiatic acid; (B) profile of *C. asiatica* extract, (C) wedelolactone, (D) profile of *Wedelia trilobata* extract

**Supplementary Figure 2**


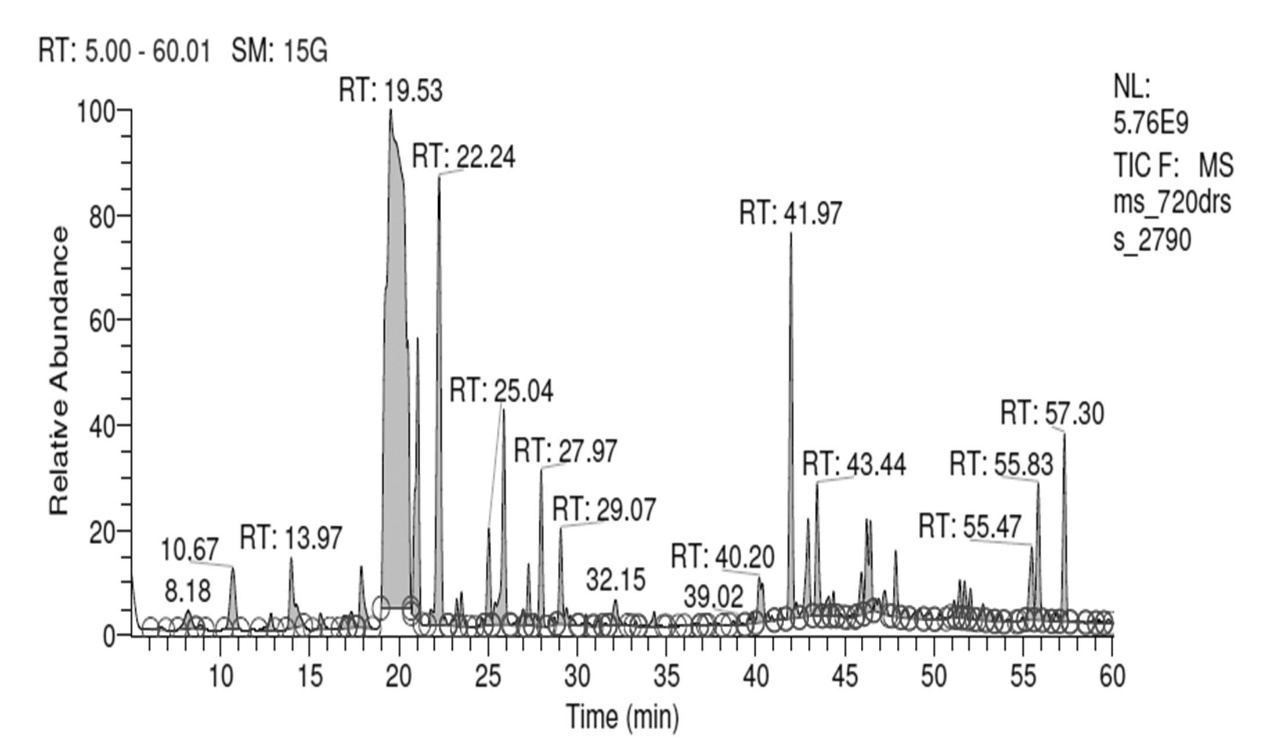


**Figure S2.** Gas chromatography profile of *Eucalyptus citriodora* oil showing major markers


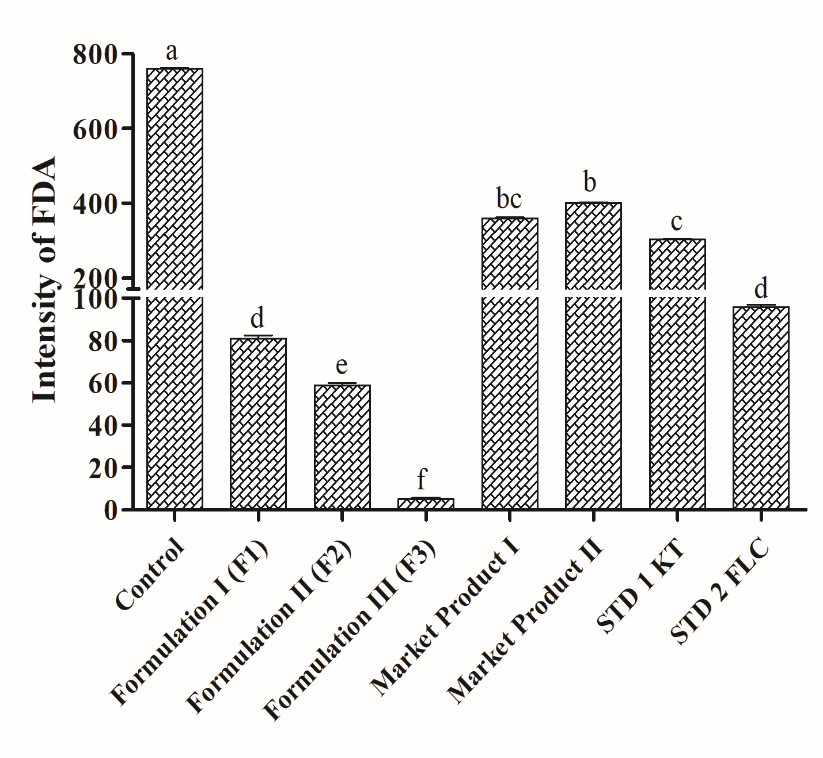

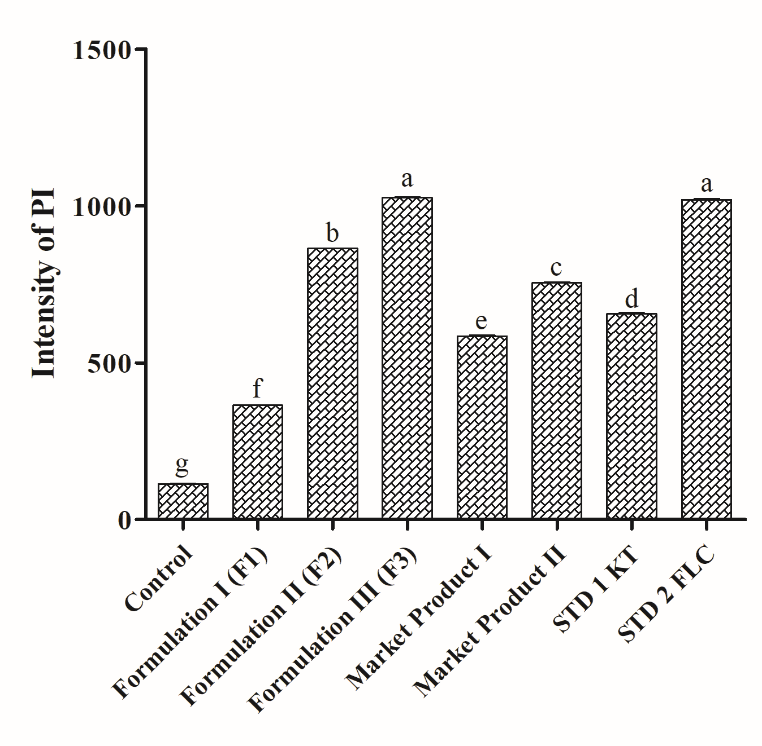
**Supplementary Figure 3**

**B**

**A**

**Figure S3.** Average florescence intensity of FDA/PI of *M. furfur* spores after treatments. Means followed by the same letter(s) within the column are not significantly different according to Tukey’s multiple comparison test (P < 0.05).
