# Supplementary material for: A synergistic herbal formulation targeting Malassezia furfur and Staphylococcus epidermidis for effective dandruff management
Source: Front Microbiol. 2025 Nov 21;16:1654658. doi: 10.3389/fmicb.2025.1654658 (PMC12679390; doi:10.3389/fmicb.2025.1654658)
Supplement: Supplementary file 3 [file Supplementary_file_3.docx]

**Table S1. Physicochemical analysis of plant samples**

| **Plant samples** | **Physicochemical parameters^*^** | | | |
| --- | --- | --- | --- | --- |
|  | **Alcohol soluble extractive**  **Value (%)** | **Water soluble extractive**  **Value (%)** | **Total ash**  **Value (%)** | **Acid insoluble ash Value (%)** |
| ***C. aciatica*** | 13.69 | 29.12 | 12.20 | 0.315 |
| ***W. trilobata*** | 14.25 | 10.02 | 9.5 | 8.22 |

**^*^n=3, values are mean±S.D.**

**Table S2. Physicochemical characteristics of EC essential oil**

| **Sample** | **Characteristics** | **Observations** |
| --- | --- | --- |
| ***Eucalyptus citriodora* oil** | Solubility | Petroleum ether, hexane, alcohol |
|  | Color | Pale yellow |
|  | Olfactory character | Lemon grass like |
|  | pH | 7.0 |

**Table S3. Phyto-molecules identified from gas chromatography of EC oil**

| **Rt** | **Compound Name** | **Chemical Composition (%)** |
| --- | --- | --- |
| 10.67 | β-Pinene | 1.52 |
| 13.95 | Eucalyptol | 1.64 |
| 17.86 | 5-Carcanol | 1.29 |
| **19.51** | **Isopulegol** | **42.26** |
| **21.06** | **p-Methan-3-one** | **4.79** |
| **22.39** | **Citronellol** | **8.54** |
| 25.06 | Epoxy-Linalool oxide | 1.31 |
| **25.89** | **Citronella formate** | **3.64** |
| 27.32 | Caryophyllene | 2.78 |
| 28.01 | Cyclohexanol | 2.29 |
| 29.09 | Mentho glycol | 1.70 |
| 40.38 | Tetrapenol | 1.21 |
| 47.87 | I-Hepta triacontanol | 0.87 |

**Table S4. Antimicrobial activity of selected herbal leads against *M. furfur* and *S. epidermidis***

| **Table S4.1 Antimicrobial activity of *C. asiatica*** **against *M. furfur*** | | | | |
| --- | --- | --- | --- | --- |
|  | **20mg/ml** | **40mg/ml** | **60mg/ml** | **80mg/ml** |
| ***C. asiatica*** | 9±0.0 | 12.3±.57 | 12.7±.6 | 13.3±.58 |

| **Table S4.2 Antimicrobial activity of *C. asiatica*** **against *S. epidermidis*** | | | | | | | |
| --- | --- | --- | --- | --- | --- | --- | --- |
|  | **20mg/ml** | | | **40mg/ml** | **60mg/ml** | | **80mg/ml** |
| ***C. asiatica*** | 10.67±.58 | | | 12.3±1.5 | 13±1 | | 14.3±1.15 |
| **Table S4.3 Antimicrobial activity of *Wedelia* sp.** **against *M. furfur*** | | | | | | | |
|  | | **20mg/ml** | **40mg/ml** | | **60mg/ml** | **80mg/ml** | |
| ***W. trilobata*** | | 9.33±.57 | 11±1 | | 10.33±.6 | 12.3±.58 | |

| **Table S4.4 Antimicrobial activity of *Wedelia* sp.** **against *S. epidermidis*** | | | | |
| --- | --- | --- | --- | --- |
|  | **20mg/ml** | **40mg/ml** | **60mg/ml** | **80mg/ml** |
| ***W. trilobata*** | 11±1 | 12±1 | 11.67±.58 | 12.3±.57 |

*** Inhibition zone (in mm) values are mean of three independent replicates with ± standard error (SE)**

**Table S5. Antimicrobial activity of EC oil**

| **Table S5.1 Antimicrobial activity of EC oil against *M. furfur*** | | | | |
| --- | --- | --- | --- | --- |
|  | **1** | **2** | **3** | **Average±S.E.** |
| **3%** | NSI | NSI | NSI | NSI |
| **5%** | NSI | NSI | NSI | NSI |
| **8%** | 20 | 20 | 21 | 20.33±0.3^d^ |
| **10%** | 35 | 40 | 33 | 36± 2.08^a^ |
| **12%** | 40 | 38 | 34 | 37.3± 1.8^a^ |
| **14%** | No Growth | No Growth | No Growth | No Growth |
| **KT 50mg** | 28 | 26 | 30 | 28.3±1.5^b^ |
| **FLC 50mg** | 26 | 29 | 25 | 26.7±2.1^c^ |

* **NSI=No Significant Inhibition (<10mm inhibition)**

** **Inhibition zone (in mm) values are mean of three independent replicates with ± standard error (SE)**

***** Means followed by the same letter(s) within the column are not significantly different according to Tukey’s multiple comparison test (P < 0.05).**

| **Table S5.2. Antimicrobial activity of *E. citriodora* oil against *S. epidermidis*** | | | | |
| --- | --- | --- | --- | --- |
|  | 1 | 2 | 3 | Average±S.E. |
| **3%** | NSI | NSI | NSI | NSI |
| **5%** | NSI | NSI | NSI | NSI |
| **8%** | 25 | 21 | 26 | 20.33±0.3^b^ |
| **10%** | 40 | 37 | 39 | 38.6± 0.9^a^ |
| **12%** | No Growth | No Growth | No Growth | No Growth |
| **14%** | No Growth | No Growth | No Growth | No Growth |
| **KT 50mg** | 13 | 14 | 14 | 13.6± 0.33^c^ |
| **FLC 50mg** | 13 | 15 | 14 | 14±0.6^c^ |

* **NSI=No Significant Inhibition (<10mm inhibition)**

** **Inhibition zone (in mm) values are mean of three independent replicates with ± standard error (SE)**

***** Means followed by the same letter(s) within the column are not significantly different according to Tukey’s multiple comparison test (P < 0.05).**

**Table S6: Binding energy of phyto-molecules against LDM**

| **Sr. No.** | **Phytomolecules/Standards** | **dG (Kcal/mol)** |
| --- | --- | --- |
|  | Beta-caryophyllene | -8.9 |
|  | Beta-pinene | -6.4 |
|  | Citronelol | -5.7 |
|  | Citronellyl formate | -5.8 |
|  | Cyclohexanol | -4.9 |
|  | Epoxylinalool oxide | -6.1 |
|  | Eucalyptol | -6.8 |
|  | Isopulegol | -6.5 |
|  | p-menthan-3-one | -6.5 |
|  | Fluconazole | -8.2 |
|  | Ketoconazole | -10.3 |
|  | Zinc pyrithione | -5.2 |
